# Capacities for the Risk Assessment of GMOs: Challenges to Build Sustainable Systems

**DOI:** 10.3389/fbioe.2018.00040

**Published:** 2018-04-05

**Authors:** Danilo Fernández Ríos, Clara Rubinstein, Carmen Vicién

**Affiliations:** ^1^Facultad de Ciencias Exactas y Naturales, Universidad Nacional de Asunción, Asunción, Paraguay; ^2^ILSI Argentina, Buenos Aires, Argentina - Monsanto Argentina, Buenos Aires, Argentina; ^3^School of Agriculture, University of Buenos Aires, Argentina - ILSI Argentina, Buenos Aires, Argentina

**Keywords:** risk assessment, capacity building, biosafety regulatory systems, problem formulation, collective action

## Abstract

The need for functional risk assessment bodies in general, and in the biosafety field in particular, demands continued efforts and commitment from regulatory agencies, if results that are sustainable in time are to be achieved. The lack of formal processes that ensure continuity in the application of state of the art scientific criteria, the high rotation in some cases or the lack of experienced professionals, in others, is a challenge to be addressed. Capacity building initiatives with different approaches and degrees of success have been implemented in many countries over the years, supported by diverse governmental and non-governmental organizations. This document summarizes some capacity building experiences in developing countries and concludes that risk assessors taking ownership and regulatory authorities fully committed to developing and retaining highly qualified bodies are a *sine qua non* to achieve sustainable systems. To this end, it is essential to implement “in-house” continuing education mechanisms supported by external experts and organizations, and inter-institutional cooperation. It has to be noted that these recommendations could only be realized if policy makers understand and appreciate the value of professional, independent regulatory bodies.

## Introduction

Investments in the establishment of functional biosafety regulatory systems and in periodical revisions for the continued improvement and modernization of existing ones are necessary if the benefits of agricultural biotechnology are to be realized.

Transparent and efficient regulatory systems with clearly defined criteria and procedures to process product applications and that can make timely and predictable science based decisions is a precondition for sustainable investments in research and development, technology transfer and product deployment.

The regulation of genetically modified (GM) crops has been criticized as a constraint to innovation in agriculture, particularly by public sector developers, largely because of the high costs of generating the data required globally by regulatory authorities for commercial approvals. In some countries, this is further complicated by inadequate local capacities to monitor compliance with biosafety measures through timely inspections, guidance and advice. In spite of these criticisms, it is agreed that predictable, consistent regulatory systems can be a powerful stimulus for investments in agricultural innovation.

Capacity building is a critical factor for the development and implementation of functional biosafety regulatory systems and requires a sustained commitment of human and material resources.

Numerous initiatives with different approaches have been implemented in many countries for over 20 years with varied degrees of success, supported by diverse governmental and non-governmental organizations. These programs were primarily aimed to build in-country capacities but also enabled the active participation of country experts in international fora. In fact, inclusive discussions at the regional and international levels are critical to develop consensus on scientific criteria, conceptual tools and common standards for evidence based risk assessment and regulations, ultimately facilitating greater harmonization among countries and regions (OECD, 2005; Bartholomaeus et al., 2015).

A shortcoming of many of these capacity building programs, however, is that while they do help with the understanding of the basic scientific criteria and internationally accepted approaches to risk assessment, the analysis of reference documents and the practical use of these tools to case studies, they do not provide the continued support needed to establish, adopt and then implement these systems in country over time.

Risk assessment is a dynamic, scientific exercise that requires significant technical capacity. The problem formulation methodology is currently considered the starting point, at which the appropriate characterization of the problem is made. The identification of available sources of information and the need for additional evidence to respond to risk hypotheses, subsequently help characterize the risk involved and make a decision about its acceptability and eventually propose risk management or mitigation measures.

In most countries there are not formal specialization options, therefore, only practice and experience make professional risk assessors and this is a lengthy process that may take 3 to 5 years. The lack of “in-house” formal processes to train and update risk assessors on the evolving scientific criteria, the high rotation in some cases, or the lack of experienced professionals and resources in others, can be challenging.

This is especially true in developing countries, but is also a challenge for some developed, mature systems, as discussed in a recent reflection paper for the European Food Safety Authority (EFSA) case (Deluyker, 2017). The author points out to the difficulties to ensure the continued availability of qualified experts for the Scientific Panels (suggests to extend the appointment period of panel members to 5 years instead of the current 3-year term and to be renewable for an extra term). According to the author, another challenge for EFSA is “how to ensure that the EU maintains adequate future expert capacity for scientific assessments. This requires on the one hand that training is offered and on the other that there are adequate opportunities to gain experience.”

Workshops, symposia, courses and conferences can be informative and are valuable to raise awareness or catalyze discussions that may aid in the development of strategic programs. However, only the continued engagement with the practice of risk assessment of those who are directly tasked with the responsibility of regulatory oversight, leads to the formation of professional, expert bodies.

The purpose of this paper is to discuss some experiences on capacity building efforts to support regulatory systems in different countries, in order to learn from the past and bring up some ideas for the development of self-sustainable systems in the future. These experiences were shared at a special panel during the 14th ISBGMO meeting that took place in Guadalajara (Mexico) in June, 2017.

A recent, 3 year collaborative program implemented in Paraguay will be discussed in detail regarding the outcomes and the challenges faced during and after the process, as a leading case with common features with several other cases shared at the session. Additional contributions by panel members will be also summarized as examples of capacity building experiences in their respective countries.

Recommendations resulting from this session and similar ones that can follow may contribute to improve and make capacity building programs more efficient and self-sustainable.

## Partnership for biosafety risk assessment and regulation: a collective action in paraguay

Activities with agricultural biotechnology were first regulated in Paraguay in 1997, followed by other legal instruments. The more recent, a Decree from 2012, created the National Agricultural and Forestry Biosafety Commission (CONBIO) with the mission to assess, analyze and issue recommendations on all matters related to the introduction, field trials, pre-commercial and commercial release, and other intended uses of GM crops (Ministry of Agricultures and Livestock, 2017). CONBIO identified the need to update the existing framework, so that it would keep up with the evolution of scientific knowledge and experience with genetically engineered technologies.

As part of this process, the Paraguayan Ministry of Agriculture joined the “Partnership for Biosafety Risk Assessment and Regulation” through the signature of a Memorandum of Understanding between the National Agricultural and Forestry Commission and the International Life Science Institute (ILSI) Research Foundation, in November 2012.

The purpose of the Program was to contribute to the improvement of technical capacity for biosafety risk assessment and regulation, so as to further strengthen institutional governance of agriculture biotechnology in Paraguay. Activities were framed within the Partnership for Biosafety Risk Assessment and Regulation, a global project led by ILSI Research Foundation and funded by the World Bank. The purpose of this partnership was *to strengthen the technical capacity of developing country stakeholders in regards to biosafety risk assessment and regulation* (ILSI Research Foundation, 2015).

Importantly, the program plan for Paraguay was designed in response to feedback received in previous meetings with the Paraguayan government representatives to assess current capacities and identified needs, as well as with inputs received in interviews with researchers, regulators, farmers and other stakeholders interested in agricultural biotechnology. A close follow-up along the implementation phase of the program, allowed incorporating suggestions and recommendations from participants so adjustments could be made accordingly.

One of the factors that contributed to this program's success was the collective action, as researchers, regulators, professionals and technicians from the CONBIO, ILSI Research Foundation and ILSI Argentina worked closely and fruitfully in the implementation and follow-up of the program. The program also benefited from the active collaboration of other organizations such as the National University of Asunción and the Argentine Council for Information and Development of Biotechnology (Argenbio), IICA's office in Paraguay (Interamerican Institute for Agricultural Cooperation) and the Institute of Agricultural Biotechnology in Paraguay (INBIO).

Broadly, activities included seminars and workshops on agricultural biotechnology targeted to a wider, interested audience and specific working sessions focused on regulators, scientists and graduate students, with in depth discussions of risk assessment concepts and tools. These specific actions were implemented for those professionals directly involved in risk assessment activities, using a hands-on methodology. Participants included professionals from the Ministries of Agriculture and Livestock, Public Health and Social Welfare, Industry and Commerce, the National Service of Animal Quality and Health, the National Service of Plant and Seed Quality and Health, the National Institute of Forestry, the Paraguayan Institute for Agricultural Technology, the Secretariat of the Environmental and the National University of Asunción.

Seven seminars and workshops were conducted by 18 expert trainers along the entire program, both in the classroom and during visits to confined field trials. In addition to the analysis of six case studies specially developed, numerous documents and tools were provided online through the project's website. A series of e-Learning courses were also developed by the Research Foundation, available in Spanish, as a follow up tool (ILSI Research Foundation, 2017). A guide for the management of confined field trials with GM plants was an additional product of this program, responding to a request from participants.

The unifying concepts used in the risk assessment of GM crops (both environmental and food/feed safety aspects), based on Problem Formulation (Wolt et al., 2010; Garcia-Alonso, 2013), were instrumental to provide a solid scientific basis to the decision-making process.

The transition from the so called “checklist” approach to one based on problem formulation was not a trivial undertaking for CONBIO's members, as the learning curve of the regulators and the time needed to adjust are generally underestimated. The main difficulty in this aspect, was to integrate the problem formulation process within the main evaluation strategy and identifying protection goals (Garcia-Alonso and Raybould, 2014), as these should be set by federal level laws and policies, which in this case did not specify which these were.

It was also difficult for risk assessors, usually trained as researchers, to adjust to a different way of analyzing information based on regulatory science criteria and examining dossiers as a source of data that respond to risk hypotheses, rather than as an academic paper (Klimisch et al., 1997)^1^.

In part as a result of this program and thanks to a deeper understanding of the scientific underpinnings of the biosafety oversight, it was possible to develop science based risk assessment instruments in Paraguay (Soerensen et al., 2014). In fact, after the completion of the program, CONBIO members issued a set of science based guidelines and application forms for experimental release in confined field trials and for the commercial authorization of GM crops, formalized through a Resolution (N° 27/2015) of the Ministry of Agriculture and Livestock of Paraguay.

Currently, the first confined field trials on private land are being carried out in Paraguay^2^. For these trials to be possible technicians had to be trained to assess the fields and monitoring systems had to be established to ensure compliance with the confinement conditions required and with adequate chain of custody processes for transgenic seeds. However, additional capacities have to be put in place as the number of trials grows.

Since its completion in 2015, the program partners have implemented periodical follow-up meetings, special sessions to discuss particular topics or to update risk assessors on new information and developments, share publications, etc.

The Partnership program also fostered open discussions among participants and with other stakeholders, contributing to enhance the level of participation of the representatives of Paraguay at regional and international meetings like OECD's working groups and other fora.

In spite of the program success in terms of capacity building, CONBIO still faces numerous difficulties. The fact that its members are not fully dedicated to risk assessment, but have other responsibilities as part of their jobs, turns the assessment into a lengthy process. Besides, members change with a certain frequency, further complicating the situation. Importantly, experts/advisors appointed to CONBIO by member institutions are experts in their fields but very seldom in risk assessment, resulting often in discussions and concerns that could be avoided with a dedicated group specialized in risk assessment. To this end, staff positions, formal trainings, hands-on experiences, inter-agency collaboration and the continued practice of biosafety assessment are key, all of which will only be possible if pertinent authorities commit to provide the needed resources.

## Experiences and learnings from capacity building efforts in other countries

The panel on “Capacities for the Risk Assessment of GMOs: challenges to build sustainable systems,” also included presentations of experiences from Argentina, Brazil, Kenya, Nigeria, South Africa, Uganda and other African countries, supported by organizations like the Food and Agricultural Organization of the United Nations (FAO), the Brazilian Agriculture Research Corporation (EMBRAPA), the Ministry of Agroindustry (Argentina), Michigan State University, ILSI Research Foundation, the International Centre for Genetic Engineering and Biotechnology (ICGEB) and the NEPAD (New Partnership for Africa's Development) African Biosafety Network of Expertise.

Martin Lema and Agustina Whelan from the Biotechnology Directorate (Ministry of Agroindustry) of Argentina, shared their experience with training programs as trainers. In particular, in the activities of the National Advisory Committee on Agricultural Biotechnology (CONABIA) as a FAO Centre of Reference for biosafety of GMOs, conducting workshops and training sessions in different countries in Sub-Saharan Africa and Ecuador, among others. The importance of training trainers and “learning by teaching others” was highlighted in this presentation. In their experience, the lack of indicators to measure efficacy needs to be addressed.

Ruth Mbabazi, Marc Heijde, and Karim M Maredia shared the capacity building efforts lead by Michigan State University for research, innovation and application of biotechnology for food security in Africa. They explained that national governments and regional economic communities (RECs) in Africa are taking positive steps in building their capacities for adoption of new technologies to enhance agricultural productivity, food and nutritional security and economic growth. This has also triggered strategic and effective public-private partnerships for translating research into practice.

Agricultural biotechnology capacity building experiences in Africa were summarized, detailing the respective roles and contributions of key continent-wide and international institutions. This presentation examined issues related to the need for technology transfer policies, practices and regulatory oversight of biotech products to enable adoption in Africa, highlighting the need to build networks to facilitate inter-country collaboration. Important challenges to be considered: the high turnover of risk assessors and difficulties to measure efficiency of capacity building initiatives.

John Teem and Libby Williams (ILSI Research Foundation) presented their e-Learning platform as a sustainable and interactive resource. While in-person workshops and meetings are an ideal way to provide education and training, several challenges can make this traditional style of capacity building increasingly difficult, including limited resources and travel constraints. By being cost-effective, interactive and accessible, e-Learning courses can be used to complement face-to-face trainings to achieve optimal learning outcomes and also be a continuing education tool.

This presentation was complemented by a capacity building case study that involved the National Biosafety Authority (NBA) of Kenya utilizing e-Learning courses developed by the Research Foundation to share biosafety information in a resource-efficient format. These resources have been translated into other languages besides English and include open access courses related to biosafety, biotechnology and food safety.

Along these lines, Dennis Ndolo, Michael Wach, Patrick Rüdelsheim and Wendy Craig introduced a curriculum-based approach to teaching biosafety through e-Learning developed by the International Centre for Genetic Engineering and Biotechnology (ICGEB). They emphasized that working in biosafety capacity enhancement incorporating approaches into activities, such that their impact becomes sustainable once funding has been depleted, can be a truly everlasting task.

Many training efforts face the limitation of one-off events: they only reach those people present at the time. However, beyond the initial effort to establish the basic content, repeating capacity enhancement events in different locations is usually not economically feasible. Also the lack of infrastructure and other resources needed to support a robust training program hinder operationalizing a “train-the-trainer” approach to biosafety training.

One way to address these challenges is through the use of e-Learning courses that can be delivered online, globally, continuously, at low cost, and on an as-needed basis to multiple audiences. Crucial to the implementation of such an e-Learning program is an approach in which the courses are intentionally developed together as a cohesive curriculum. Once developed, such a curriculum can be released as a stand-alone program for the training of governmental risk assessors or used as accredited components in graduate degree programs in biosafety, at minimal cost to the government or university. Examples from the ICGEB portfolio of biosafety e-Learning courses were presented to demonstrate these key features.

Deise Maria Fontana Capalbo from the Brazilian Agriculture Research Corporation (EMBRAPA)—Environment, shared Brazilian capacity building experiences in biosafety to support the decision-making process. The main decision body in place in Brazil is the National Biosafety Technical Commission (CTNBio), composed of 27 members and their respective alternates that hold a two-year term, renewable for up to two consecutive periods.

This presentation showed some experiences on how individuals, groups, institutions and governmental authorities acted in order to provide training and technical assistance to the decision-making bodies. There were, and still are, many types of capacity building activities in place. Different approaches incorporated a variety of forms and disciplines and many factors were taken into account (target beneficiaries, effective content according to the target audience, specific needs, integration and collaboration among the various disciplines and capacity builders). An active participation of country experts in international fora is also encouraged in Brazil.

Finally, Samuel Timpo from the NEPAD Agency African Biosafety Network of Expertise (ABNE) in collaboration with Hashini Galhena Dissanayake (Michigan State University), Joseph Guenthner (University of Idaho), Godwin Lemgo (NEPAD), and Karim Maredia (Michigan State University), introduced institutional capacity efforts to overcome systems challenges toward building functional biosafety systems in Africa. While functional biosafety systems are critical for the safety assessment of GM crops, the development of these systems in Africa are constrained by a number of factors. Key among these factors is the lack of institutional and human capacity to design and implement biotechnology regulatory frameworks that have the capability to make science-based decision on risks and benefits of various GM crops as well as provide mechanisms for inspection, monitoring and compliance.

In view of these on-going efforts, authors attempted to identify knowledge and skill gaps through a multi-stakeholder field research carried out in six countries in Africa and discussed strategies to enhance biosafety capacity. The findings highlighted the importance of continuing capacity building programs and coordinating efforts and investments as well as broadening training modules and extending to groups beyond regulators, policy makers, and scientists. Such efforts will help minimize prevalent concerns about food and environmental risk and empower stakeholders with accurate information to counter misconceptions.

## Points of agreement and recommendations

- Regulatory guidelines based on sound science and risk assessment experience assist regulators in their evaluations and well-designed capacity building programs, customized to the different realities and particular needs of each country or region, can greatly contribute to build regulatory capacity.
- Biosafety systems deal with evolving concepts and technologies and need to anticipate and adjust their procedures and requirements to these advances. Therefore, a periodical revision of regulations and operations is always necessary (Vicién and Trigo, 2017). These processes require to be accompanied by permanent training cycles for risk assessors, which consider state of the art criteria and methodologies.
- For any program to be effective, it is recommended to implement early consultations and interviews with key stakeholders as well as post-training contacts for feedback and follow-up support. It is good practice to customize the programs.
- As much as these programs can give support, it will always be from the outside. Intra-agency processes must be in place in order to become self-sustainable.
- The panel concurred on the barriers to achieve sustainable systems and drew attention to the high rotation of risk assessors in some countries. The lack of dedicated professional regulators, specialized in risk assessment, is a key factor.
- To this point, the need for professional programs (specializations, degrees, masters programs) was mentioned. Academic programs are lacking in most countries and would provide a formal context to develop capacities, also offering new career development opportunities to university graduates.
- Regarding technological tools (as online courses, etc.) as much as they can help, they have to be part of integral programs, as they do not work in isolation.
- In a risk-averse society, concepts on Risk, Biosafety, Risk Science, Problem Formulation and Regulatory Sciences should be part of University curricula and even of High School programs, to educate citizens and build trust in regulatory bodies.
- Governmental commitment to support the establishment of self-sustainable systems is also essential in order to give continuity to regulatory systems, without depending on sporadic funding. To this end, it is basic to educate and engage political leaders and decision makers, on the importance of having professional, expert regulatory agencies.
- The development of metrics and indicators to assess the effectiveness of these efforts is also recommended, as this can facilitate further improvements and a better use of resources often times provided by governments, universities or international organizations.
- These metrics should be able to assess the degree of maturity of the systems, the efficiency of training efforts and their sustainability. Potential indicators can be developed around the general improvement of the decision making processes, the quality and robustness of decision documents, the implementation of consistent, transparent assessment criteria, the application of tools shared in trainings, the intra-agency processes put in place to train new regulators and keeping staffs up to date, the use of inter-agency consultations /collaborations, etc.
- Panel members also agreed on the importance of personal experience, as well as teaching others, as great learning experiences. The common message was around the value of partnerships.
- In summary, it takes time to design regulatory frameworks and to develop expertise to put them into practice and conduct periodical updates. This is an everlasting process and as technologies and frameworks evolve, regulators need to be prepared to keep up with these changes in a dynamic and continuous cycle.
- As valuable as the efforts made by diverse organizations might be, these will fail to build sustainable systems without country policy decisions to support the development of dedicated, professional and transparent regulatory bodies that can focus on the high responsibility of protecting environmental and human health.

This has been a first attempt to share experiences and identify barriers to sustainable systems. It would be desirable to follow up on these discussions in order to put some of these recommendations (like formal training opportunities or the development of metrics), into practice.

## Author contributions

All authors participated in the drafting of this paper as individual experts in their fields, and the authors are solely responsible for the contents. Any views expressed in this paper are the views of the authors and do not necessarily represent the views of any organization, institution, or government with which they are affiliated or employed.

### Conflict of interest statement

The authors declare their affiliations and employers in the authors list. This work has not received specific funding. ILSI is a non-profit network of scientific, tripartite institutions that advocate for the use of science for the improvement of human health and well-being and safeguards the environment.

## Footnotes

^1^Regulatory science is a scientific discipline that poses risk hypotheses derived of the problem formulation step and considers policy protection goals. It generates data using standardized protocols, validated methodologies and quality assurance systems to ensure data integrity

^2^Field trials for GE crops in Paraguay have been conducted almost exclusively by IPTA (the Paraguayan Institute for Agricultural Technology). This policy was recently modified to offer developers broader options.
